# Pushing the boundaries of laparoscopic myomectomy: a comparative analysis of peri-operative outcomes in 323 women undergoing laparoscopic myomectomy in a tertiary referral centre

**DOI:** 10.1186/s10397-017-1025-1

**Published:** 2017-11-13

**Authors:** Rebecca Mallick, Funlayo Odejinmi

**Affiliations:** Department of Gynaecology, Barts Health NHS Trust, Whipps Cross University Hospital, London, E11 1NR UK

**Keywords:** Laparoscopic myomectomy, Outcomes, Complications

## Abstract

**Background:**

The aim of this study was to analyse the demographic data and peri-operative outcomes of women undergoing a laparoscopic myomectomy and assess what factors, if any, precluded using the laparoscopic approach.

**Methods:**

A single surgeon observational study of 323 patients undergoing a laparoscopic myomectomy was undertaken. Data was collected prospectively over a 12-year period and analysed using SPSS. Surgical outcomes included operating time, estimated blood loss, conversion to laparotomy, intraoperative and postoperative complications and duration of inpatient stay.

**Results:**

A total of 323 patients underwent a laparoscopic myomectomy over the 12-year period. The majority of fibroids removed were intramural (49%) and subserosal (33%). The mean size of fibroids removed was 7.66 ± 2.83 (7.34–7.99) cm, and the mean number was 4 ± 3.62 (3.6–4.39), with the greatest being 22 removed from a single patient. Average blood loss was 279.14 ± 221.10 (254.59–303.69) ml with mean duration of surgery and inpatient stay recorded as 112.92 ± 43.21 (107.94–117.91) min and 1.88 ± 0.95 (1.77–1.99) days, respectively. No major intraoperative complications were noted, and the conversion to laparotomy rate was 0.62%. All histology following morcellation was benign. Over the 12-year period despite increasingly large and more numerous fibroids being tackled, increasing experience resulted in a simultaneous reduction in overall blood loss, operating time and duration of inpatient stay.

**Conclusions:**

Laparoscopic myomectomy is a safe and efficacious procedure that should be considered the gold standard surgical treatment option for fibroids. With experience, the procedure can be undertaken with minimal complications, a low risk of conversion to laparotomy and early discharge from hospital, even in cases of large and multiple fibroids that historically would have required the open approach. This allows even the most complex of cases to now benefit for the advantages of the minimal access approach.

## Background

Uterine fibroids remain the commonest benign tumour encountered in the female population. They affect around 20–25% of women with symptoms ranging from heavy menstrual bleeding and pressure to subfertility. The clinical features and treatment options offered are largely dependent on the type, number and position of the fibroids, and historically, the surgical treatment of choice was an open myomectomy or abdominal hysterectomy. However, many women, especially those of reproductive age, prefer more conservative uterine-preserving techniques, and the minimal access route offers significant benefits both in terms of recovery and future fertility [1–3]. These benefits are now well established in the wider literature, and the minimal access route should generally be considered the gold standard surgical treatment for such women. But is there a limit to what can be performed laparoscopically and what are the risks? Are there specific demographic features that can affect the surgical outcome and can help guide us when counselling patients and choosing the safest surgical procedure? Since the first laparoscopic myomectomy, described by Kurt Semm in 1979 [4], there have been significant technological advances and increasing expertise in minimal access surgery and the previously considered contra-indications to laparoscopic myomectomy [5, 6] may no longer exist in experienced hands. The aims of this study were to analyse the demographic data and peri-operative outcomes of 323 women who underwent a laparoscopic myomectomy over a 12-year period in a tertiary referral centre in London and compare our findings to the published literature.

## Methods

This is a prospective observational study of 323 patients who underwent a laparoscopic myomectomy from March 2004 to November 2016. All patients were operated on by a single surgeon at Whipps Cross University Hospital Trust. The only exclusion criteria was uterine size greater than 28 weeks size limiting access to the pelvis. A standardised technique was used; initial entry was through an intra-umbilical incision or palmer’s point in cases where the uterine size was more than 14 weeks, with two 5 mm ancillary lateral ports and a suprapubic port. For haemostasis, 800 μg misoprostol was administered rectally and vasopressin injected intra-myometrially unless contraindicated. For uterine manipulation, a ClearView™ (Clinical Innovations) uterine manipulator was used to achieve the optimum uterine position. For most cases, the Harmonic™ (Ethicon) scalpel was used, but more recently, alternative ultrasonic energy devices such as Thunderbeat™ (Olympus) have been introduced. A 5 mm myoma screw and grasping forceps were used for traction and counter traction. Initially, the resulting defects were closed in two or three layers as required using polyglactin sutures for the myometrium and monofilament sutures used for the serosa; however, more recently, self-retaining sutures such as V-loc™ (Covidien) and Stratafix™ (Ethicon) have been introduced. For adhesion prevention, due to the discontinuation of Sprayshield™ (Covidien), Interceed™ (Ethicon) has been used since 2014. Myomas were removed by electro-mechanical morcellation through the suprapubic port, currently without the use of containment/morcellation bags, and sent for histology. Initially, we used the Johnson and Johnson Ethicon morcellator; however, when it was withdrawn, we changed to the RotoCut morcellator (Stortz), and more recently, we have been using the LiNA morcellator.

All patients were assessed for suitability pre-operatively and had radiological fibroid mapping using either ultrasound or MRI. Patients were given written information regarding their procedure and counselled preoperatively about their recovery and anticipated discharged within 24 h. Also, following the concerns regarding the possibility of dissemination of malignant cells during the morcellation process [7], we considered the information provided by national and international societies [8, 9] and devised a leaflet explaining the risks of morcellation. This leaflet contained not only information about the potential risk of dissemination of malignancy and the risk of upstaging a leiomyosarcoma, but also the possible risks of visceral injury. All patients were also given antibiotics prophylaxis at induction of anaesthesia and appropriate thromboprophylaxis.

Patient demographics (age, BMI, parity), procedure details and patient outcomes were collected prospectively from 2004 onwards on an Excel spreadsheet and entered immediately following each procedure and on discharge from hospital. The data was analysed using SPSS (version 22). The *t* test was used for the comparison group analysis, but if the data failed the homogeneity assumption (Levene’s test), a Mann–Whitney test was undertaken. A *p* value of 0.05 was considered significant. Formal ethical approval was not required as this was an evaluation of ongoing surgical practice.

## Results

**Fig. 1:**
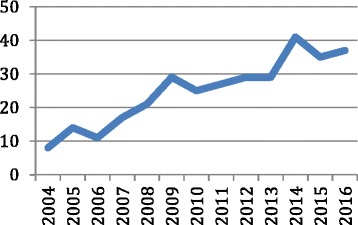
Laparoscopic myomectomies per year

Three hundred twenty-three patients underwent a laparoscopic myomectomy over the 12-year period. There was almost a fivefold increase in the number of laparoscopic myomectomies performed in 2016 compared to 2004 (Fig. 1). Patient demographics are summarised in Table 1. 40.3% had undergone previous abdominal surgery with 27.6% undergoing a previous laparoscopic procedure and 12.7% undergoing a laparotomy.

**Table 1:** Patient demographics

Variable	Value
Age (years)	38 (24–56)
BMI	26.5 (16–46)
Parity	0 (0–6)
Ethnicity	
African/Afro-Caribbean	196 (60.7%)
Asian	43 (13.3%)
Caucasian	84 (26%)
Indications for surgery	
Bleeding	179 (55.2%)
Pain/pressure	77 (23.8%)
Infertility	68 (21%)

Data presented as median (range) or absolute number (%)

A total of 1921 fibroids were removed with the size of the dominant fibroid ranging from 2 to 20 cm (mean 7.6 cm). The majority of fibroids removed were intramural (49%) and subserosal (33%) and less frequently submucosal (17%) and pedunclulated (1%). With regards to the position of the fibroids, the majority were posterior (40%), fundal (25%) and anterior (20%) with less common sites including broad ligament (9%), lateral (3%) and cervical (3%). Thirty-one percent of patients had a single fibroid removed, and the remaining 69% required multiple fibroid removal. The mean number of fibroids removed was 4, with the greatest being 22 removed from a single patient. Average blood loss was 279.14 ml with mean duration of surgery and inpatient stay recorded as 112.92 min and 1.88 days, respectively. Operative features are summarised in Table 2.

**Table 2:** Operative features

Variable	Value
Largest fibroid removed (cm)	7.66 ± 0.165 (7.34–7.99)
Number of fibroids removed	4 ± 0.202 (3.6–4.39)
Weight of fibroids removed (g)	216.28 ± 12.127 (192.42–240.14)
Blood loss (ml)	279.14 ± 12.477 (254.59–303.69)
Duration of surgery (min)	112.92 ± 2.533 (107.94–117.91)
Inpatient stay duration (days)	1.88 ± 0.055 (1.77–1.99)

Data presented as mean ± standard error of mean (95% CI)

Twenty-three percent of patients had co-existing endometriosis, which was excised.

No major intraoperative complications were noted. Two procedures were converted to a mini laparotomy, one due to technical difficulties in closing the uterine incision and the other due to anaesthetic concerns with the patient’s ventilation, giving a conversion rate of 0.62%. Nine patients required post-operative blood transfusions (2.79%). Seven patients had documented post-operative complications (2.17%); one patient returned to theatre on day 2 due to intra-abdominal bleeding, which was managed by a laparotomy and re-suturing. Two patients had post-operative urinary retention, and four patients developed port site hernias.

All specimens were removed using electo-mechanical morcellation, and in all cases, the histology was reported as benign. There were histological variants described and these included apoplectic myoma, adenomatoid tumour and symplastic tumour. These tumours are also benign and do not need any follow-up; however, one of the patients, who had an atypical myoma, opted for a laparoscopic hysterectomy and required no further treatment.

Between the onset of the study to January 2010, out of the 41% of patients who had surgery for infertility, 56% had conceived after surgery.

When comparing the number of fibroids removed and operative outcomes, there was a significantly greater blood loss and operating time in patients having multiple fibroids removed compared to those having a single myomectomy.

Fibroids over 9 cm also had a significantly greater blood loss and operating time when compared to those less than 8 cm. Both age and BMI did not appear to have any significant impact on operative outcomes (Table 3).

**Table 3:** Fibroid and patient factors impacting operative outcomes

	Single myomectomy	Multiple myomectomy	*p* value
Blood loss (ml)	218.5	308.45	0.001
Operating time (min)	85.6	125.43	< 0.001
Inpatient stay (days)	1.81	1.9	0.437
	Fibroid < 8 cm	Fibroid ≥ 9 cm	*p* value
Blood loss (ml)	121.34	192.03	< 0.001
Operating time (min)	105.30	130.12	< 0.001
Inpatient stay (days)	1.799	1.989	0.127
	BMI < 29.9	BMI > 30	*p* value
Blood loss (ml)	272	278	0.837
Operating time (min)	112	114	0.765
Inpatient stay (days)	1.85	1.64	0.062
	Age < 40	Age ≥ 40	*p* value
Blood loss (ml)	259	193	0.059
Operating time (min)	114	94	0.054
Inpatient stay (days)	1.9	1.7	0.408

Data presented as mean

### Learning curve of laparoscopic myomectomy

**Fig. 2:**
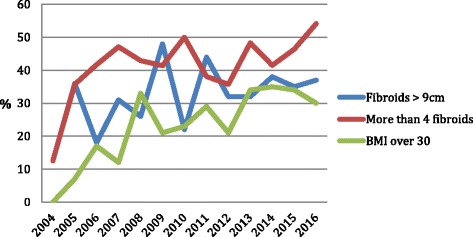
Percentage of patients with fibroids size > 9cm, greater than four fibroids and a BMI over 30 over time

**Fig. 3:**
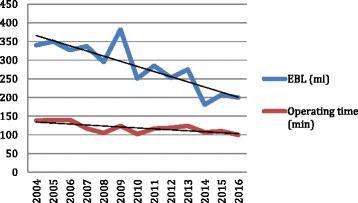
EBL (ml) and operating time (min) over time

Over the time 12-year period, the cases have become increasingly complex with larger fibroids and more numerous fibroids being tackled in women who are increasingly more obese (Fig. 2). Despite this, the increasing experience and expertise has resulted in a simultaneous reduction in blood loss and operating time (Fig. 3). Inpatient stay has been also reduced by more than half from a mean of 3.5 days to 1.3 days.

## Discussion

Since the first laparoscopic myomectomy in 1979, there has been a wealth of data published highlighting the benefits of the minimal access approach over the standard open approach. Benefits include reduced blood loss, shorter hospital stay, reduced post-operative pain and less adhesion formation, and many case series have been published worldwide [10–25] (Table 4). However, this series appears to one of the largest and the only prospective single surgeon series from the UK.

**Table 4:** A comparative analysis of case series of laparoscopic myomectomy from 1996 to 2017

	*n=*	Fibroids removed (*n*)	Largest fibroid removed (cm)	EBL (ml)	Operating time (min)	Inpatient stay (days)	Conversion rate (%)	Complication rate (%)
Dubisson 1996 [10]	213						7.5	3.8
Dessolle 2001 [11]	88	1.7 ± 0.6 (1–4)	6.2 ± 1.8 (3–11		150 ± 60 (60–300)	3.0 ± 1 (1–10)	14.8	
Landi 2001 [12]	368				100.78 ± 43.83	2.89 ± 1.3		
Malzoni 2003 [13]	144		7.8 (5–18)		95 (58–180)	2.6 (2–5)	1.39	
Malzoni 2006 [16]	982	2.23 (1–8)	6.72 ± 2.71 (1–20)		104.5 (30–360)	2.02 ± 0.61	1.29	
Rosetti 2007 [24]	332	2.23 ± 1.7 (1–8)	6.2 ± 2.7 (1–20)		124.021 ± 52.2	2.0 ± 0.57	1.51	
Yoon 2007 [15]	51	2.2 ± 1.8	9.3 ± 1.8		85.6 ± 38.9	3.2 ± 0.9	0	
Sizzi 2007 [18]	2050	2.26 ± 1.8 (1–15)	6.40 ± 2.6 (1–20)		107.71 ± 43.42	1.99 ± 0.9		2.02
Sinha 2008 [19]	505	1.85 ± 5.706	5.86 ± 3.3	90 (40–2000)	60 (30–270)			
Paul 2010 [21]	1001	1.97 (1–17)	(1–20)	248 (20–1000)	93 (20–280)	1.5 (1–5)	0.1	2.62
Tinelli 2012 [25]	235		6.6 ± 3.5 (4–10)	118 ± 27.9	84 (25–126)		0	
Sankaran 2013 [22]	125	3.69 ± 2.96 (1–15)	7.68 ± 2.95 (2–15)	339.83 ± 254.15 (50–1500)	115.9 ± 42.09 (40–200)	2.38 ± 1.09 (1–7)	1.69	
Saccardi 2014 [23]	444		7.6 ± 2.7	184.1 ± 233.5	77.2 ± 33	2.54 ± 1.1		
Sandberg 2016 [20]	731	3.54 ± 4.10		181.54 ± 342.02		0.58 ± 1.00	1.09	
Bean 2017 [17]	514	1 (1–12)	7 (1–20)	73 (5–3000)		2 (0–24)	0.4	3.5

Data presented as mean ± SD (range) or %

Historically, the laparoscopic route had been reserved for smaller and less numerous fibroids due to the advanced technical skills required, and many recommendations have been made in the literature as to the “safe” limits of laparoscopic surgery. In 1996, Dubuisson et al. [10] advised that laparoscopic myomectomy should only be considered when the fibroids are less than 8 cm and in cases where there are less than two fibroids to be removed due to the high conversion to laparotomy rates. Other authors have suggested that the presence of more than four large fibroids greater than 4 cm in diameter or a solitary fibroid greater than 10 cm in diameter should be a contraindication to the laparoscopic approach [26, 27].

More recently, due to technological advances and increasing surgical expertise, these traditional boundaries are being pushed and larger. Sinha et al. [19] published a case series in 2008 assessing myomectomies for fibroids greater than 10 cm in size and five in number. Our case series has an average fibroid size of 8 cm (range 2–20 cm), and the average number of fibroids removed was four (range 1–22), which is greater than the traditional limits and larger than the vast majority of published case series (Table 3). However, our complication rate remains low (2.17%) in keeping with other case series including those historical studies using more conservative limits [10, 17, 21]. To date, the largest laparoscopic myomectomy case series was described by Sizzi et al. [18] in 2007, which described a similarly low complication rate of 2.02% in 2050 cases, although in this case series, both the number (mean = 2.26) and size of fibroids (mean = 6.40) removed were significantly less than ours.

Historically, one of the most common complications encountered during a laparoscopic myomectomy was the need to convert to laparotomy. In one of the earliest case series, Dubusisson et al. [10] reported a conversion rate of 7.5% and attributed this to fibroid size, number and position. Furthermore in 2001, Dessolle et al. [11] reported a similarly high conversion rate of 14.8%. Over the last 10 years, with increasing experience and expertise, this has significantly dropped to reported rates in the literature of between 0 and 1.69% [15, 21, 22, 25]. We also report a low conversion to laparotomy rate of 0.62%. Reassuringly, however, our conversion rate remained low despite tackling larger and more numerous fibroids.

In our case series, the mean blood loss was 279 ml, which was comparable to the wider literature [17, 20, 21]. Nine patients required post-operative blood transfusions; however, in the majority of these cases (77%), the patients were anaemic pre-operatively and the need for transfusion was not directly related to operative blood loss. Maintaining haemostasis and bloodless enucleation are fundamental steps in a successful laparoscopic myomectomy, and a Cochrane review found a reduction in blood loss with the use of both misoprostol and vasopressin [28]. All the patients in our series were given both misoprostol 800 μg per rectum and intra-myometrial vasopressin, which helped reduce blood loss without any significant side effects.

The operating time in our case series was in keeping with the wider literature [17–19], and as one would expect, the greater the number and larger the size of fibroids, the longer the operating time, which is keeping with the findings of Sinha et al. [19]; however, this did not appear to have any significant effect on patient recovery/duration of inpatient stay. We have also found that using barbed sutures (V-loc™or Stratafix™) has helped reduce operating time, and in a recent multicenter study, the use of such barbed sutures has also been shown to significantly reduce operative blood loss [29].

The average duration of hospital stay (1.8 days) was comparable with other studies [18, 20, 21]. Sinha et al. [19] found that hospital stay increased with increasing number, size and weight of fibroids. However, our study reassuringly found no significant effect on the day of discharge in patients with large (> 9 cm) and numerous (> 4) fibroids. The reasons for this may be multifactorial, but a large factor is our extensive pre-operative counselling and well-established enhanced recovery practices.

One of the current controversies surrounding laparoscopic myomectomy is morcellation and the risk of undiagnosed sarcoma, and in April 2014, the FDA discouraged the use of electro-morcellation [7]. However, the most recent BSGE statement acknowledges the place of power morcellation and the benefits of laparoscopic myomectomy and highlights the importance of patient counselling regarding the potential risks although low [8]. Reassuringly, all the histology reported in our case series was benign. However, caution must be applied in the older age group, and to avoid the risk of undiagnosed malignancy patient selection and counselling is key. Red flag symptoms that should raise suspicion include new onset irregular or heavy vaginal bleeding, unexplained weight loss and systemic symptoms. None of the patients in this series were menopausal, and we would also not routinely offer laparoscopic myomectomy to peri-menopausal women; however, many prefer uterine conserving techniques and decline hysterectomy. In such women, detailed counselling is essential not only with regard to the potential up-staging of malignancy if they were found to have a sarcoma, but also with regard to the potential operative benefits of hysterectomy over myomectomy such as reduced blood loss, operating time and hospital stay as highlighted by Odejinmi et al. [30] when comparing myomectomy and hysterectomy in the older age group. An alternative approach is to use a morcellation bag, which contains the theoretical risk of spreading malignancy if present [31]. This practice is becoming increasingly popular, but national guidance is yet to recommend its general use. Consideration should also be given to the implementation of a national database to record outcomes following laparoscopic myomectomy and morcellation; this would also help with the counselling process and assessment of individual risk.

## Conclusions

In conclusion, our study highlights that in experienced hands, laparoscopic myomectomy is a safe and efficacious procedure with low complication and conversion to laparotomy rates even when large (> 8 cm) and numerous (> 4) fibroids are tackled, unlike the earlier studies in the wider literature. As expected, blood loss and operating time are directly related to fibroid size and number; however, large, multiple fibroids can be tackled safely without any increase in patient morbidity and length of hospital stay. Experience, ongoing self-audit of outcomes and a dedicated enhanced recovery approach are essential to the improved outcomes demonstrated in this study.
